# Impacts of Gut Bacteria on Human Health and Diseases

**DOI:** 10.3390/ijms16047493

**Published:** 2015-04-02

**Authors:** Yu-Jie Zhang, Sha Li, Ren-You Gan, Tong Zhou, Dong-Ping Xu, Hua-Bin Li

**Affiliations:** 1 Guangdong Provincial Key Laboratory of Food, Nutrition and Health, School of Public Health, Sun Yat-sen University, Guangzhou 510080, China; E-Mails: zhyujie3@mail2.sysu.edu.cn (Y.-J.Z.); zt740359815@163.com (T.Z.); xudongping1989@163.com (D.-P.X.); 2 School of Chinese Medicine, The University of Hong Kong, Sassoon Road, Hong Kong, China; E-Mail: lishasl0308@163.com; 3 School of Biological Sciences, The University of Hong Kong, Pokfulam Road, Hong Kong, China; E-Mail: ganry@connect.hku.hk

**Keywords:** gut bacteria, human health, cancer, obesity

## Abstract

Gut bacteria are an important component of the microbiota ecosystem in the human gut, which is colonized by 10^14^ microbes, ten times more than the human cells. Gut bacteria play an important role in human health, such as supplying essential nutrients, synthesizing vitamin K, aiding in the digestion of cellulose, and promoting angiogenesis and enteric nerve function. However, they can also be potentially harmful due to the change of their composition when the gut ecosystem undergoes abnormal changes in the light of the use of antibiotics, illness, stress, aging, bad dietary habits, and lifestyle. Dysbiosis of the gut bacteria communities can cause many chronic diseases, such as inflammatory bowel disease, obesity, cancer, and autism. This review summarizes and discusses the roles and potential mechanisms of gut bacteria in human health and diseases.

## 1. Introduction

The human gut mucosa consists of epithelial cells, lamia propria, and the muscularis mucosae, which is colonized by 10^14^ microbes [1]. The number of these microbes is ten times more than the human cells. Gut bacteria are important components of the microbiota ecosystem in the human gut. Commensal bacteria colonize in the gut shortly after birth and comprise approximate 1000 species, most of which are unknown species belonging to anaerobic strains [2,3]. The composition and temporal patterns of gut microbiota in infants varies widely and is very different from those in adults. Furthermore, the intestinal microbiota stabilizes to a more adult-like profile around the age of one year, usually after the introduction of solid foods [4]. In addition, the composition of the gut bacteria community in the stomach and colon is distinctive, which is mainly due to different physicochemical conditions, such as intestinal motility, pH value, redox condition, nutrients, host secretions (e.g., gastric acid, bile, digestive enzymes, and mucus), and the presence of an intact ileocaecal valve [5]. Additionally, they can be influenced by many factors, such as the use of antibiotics, illness, stress, aging, bad dietary habits and lifestyle [5,6].

Usually, gut bacteria and the host live in a commensal manner. On the one hand, they can supply essential nutrients, synthesize vitamin K, aid in the digestion of cellulose, and promote angiogenesis and enteric nerve function [7,8,9]. *Bacteroidetes* and *Firmicutes* are the main bacteria in the metabolism of undigested food remnants. They help to digest dietary fiber and polyphenols by a complex metabolic energy-harvesting mechanism, which is based on cross-feeding and co-metabolism. In return, commensal bacteria take advantage of theprotective and nutrient-rich environment of the host [10]. Yet, specialized gut bacteria perform reductive reactions such as methanogenesis, acetogenesis, nitrate reduction, and sulfate reduction [11]. On the other hand, commensal bacteria and probiotics can promote barrier integrity, and prevent antigens and pathogens from entering the mucosal tissues [12]. Besides, commensal bacteria contribute to the host defense by regulating the homeostasis of the host immune system [13]. However, gut bacteria can be potentially harmful when the gut ecosystem undergoes abnormal changes. Dysbiosis of the gut bacteria communities in patients or animal models may cause allergy, inflammatory bowel disease (IBD), obesity, diabetes, and even cancer [8,9]. The composition of gut bacteria can indicate the risk of diseases in each person [14]. Herein, this review summarizes and highlights the roles and potential mechanisms of gut bacteria in human health and diseases. Understanding of the relationship between gut bacteria and human health can be helpful for targeting new probiotic treatments and novel strategies in treating and managing a wide variety of human diseases. The literature was sought from the databases PubMed and ISI Web of Knowledge, and the references cited were mainly original articles from 2005–2014.

## 2. Gut Bacteria in Health

**Figure 1:**
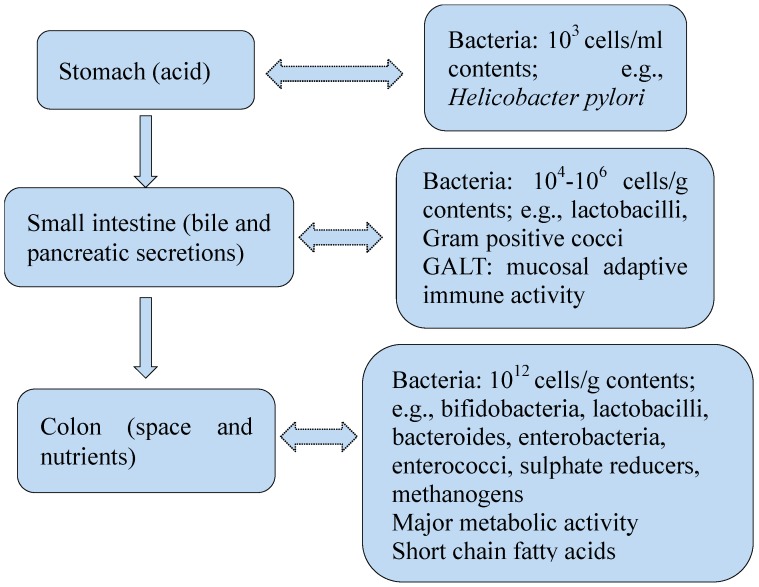
Reciprocal relationship between human gut bacteria and the host.

The main gut bacterial phyla, in the order of numerical importance, are *Firmicutes*, *Bacteroidetes*, *Actinobacteria*, *Proteobacteria*, *Verrucomicrobia* and *Fusobacteria* [15]. *Firmicutes* are gram-positive bacteria with a low G + C content, including the large class of *Clostridia* and the lactic acid bacteria, while *Actinobacteria* are gram-positive bacteria with a high G + C content, including *Colinsella* and *Bifidobacterium* spp. Lactic acid bacteria and *Bifidobacteria* are two important types of gut bacteria, which are autochthonous ones from birth or acquired from digested food. *Lactobacillus* and *Leuconostoc* spp. are the main lactic acid bacteria found in the human intestine. *Bifidobacterium* spp*.* is the predominant bacteria found among the first colonizers of newborns, and persists at a low level in adults [16]. Gut bacteria play an important role in human health, including contributing to the host gut defense system and helping the gut to maintain normal function, while its composition can be influenced by the host (Figure 1).

### 2.1. Gut Bacteria and Gut Immune System

The gut resists pathogenic bacteria through two barriers, the mechanical barrier and the immune barrier. The mechanical barrier consists of a single layer of polarized intestinal epithelial cells, the enterocytes and mucus. On the other hand, secreted immunoglobulin A (IgA), intraepithelial lymphocytes, macrophages, neutrophils, natural killer cells, Peyer’s plaques, and mesenteric lymph node compose the immune barrier. Commensal bacteria and probiotics can promote the integrity of gut barriers. Commensal bacteria contribute to the host gut defense system mainly by resisting the invasion of pathogenic bacteria and helping the development of the host immune system. Gut bacteria maintain resistance against the colonization of pathogenic bacteria by competing for nutrients and attachment sites on the mucosal surface in the colon, a phenomenon collectively known as “colonization resistance” [17]. The invasion of pathogenic bacteria is also prevented by commensal bacteria due to the reduction of the intestinal pH by the production of lactate and short-chain fatty acids (SCFAs) [9]. Another way is by producing toxic or carcinogenic metabolites to inhibit the growth or kill potentially pathogenic bacteria, together with volatile fatty acids that can inhibit the colonization of pathogenic bacteria. For example, proteolytic fermentation in the distal colon could produce toxic, carcinogenic metabolites such as bacteriocins, ammonia, indoles, and phenols by gut bacteria [18]. Lipopolysaccharides (LPSs) and peptidoglycan (PGN) components in the bacterial cell wall are two kinds of pathogen-associated molecular patterns, and they can individually or synergistically activate nuclear factor κB (NF-κB) effector and further induce the production of inflammatory cytokines such as tumor necrosis factor α (TNF-α), interleukin 1β (IL-1β) and antimicrobial peptides in the defense against foreign pathogens. Chronic stimulation of pattern-recognition receptors (PRRs) by PGN can also minimize excessive tissue injury induced by intestinal antigen-presenting cells, which can produce inhibitory cytokines such as transforming growth factor β (TGF-β) and IL-10 via nuclear oligomerization domain-2 dependent pathways [19].

Peyer’s patches, lamina propria lymphocytes, intra-epithelial lymphocytes and mesenteric lymph nodes constitute gut-associated lymphoid tissue (GALT), which is the main part of the gut immune system. Gut bacteria prime the dendritic cells (belonging to lamina propria lymphocytes) of the immune system. *L. plantarum* was suggested to regulate human intestinal epithelial tight-junction proteins and show protective effects against chemical-induced disruption of the epithelial barrier [12]. It has been found that antibody repertoire diversification occurred in GALT after birth and was stimulated by gut bacteria in sheep, cattle, pigs, and rabbits [20]. It was hypothesized this action may also occur in humans. Gut bacteria including *Bacteroides fragilis* and *Bacillus* are probably required for the normal development of GALT and mucosal immunity in all mammals. They are also required for somatic diversification of immunoglobulin (Ig) genes. Gut bacteria colonization induces a conspicuous response of the gut immune system to the production of IgA, which plays a critical role in regulation of gut bacterial communities in the small intestine. Another factor that may play an active role in the induction of local immune responses is the ILs, which may function as sensors of gut bacteria [10].

Studies of animals bred under germ-free conditions showed that germ-free animals presented morphological, structural, and functional abnormalities, including “reduced vascularity, digestive enzyme activity, muscle wall thickness, cytokine production and serum immunoglobulin levels, smaller Peyer’s patches and fewer intra-epithelial lymphocytes” [21]. Another study showed that animals received cells from germ free mice developed an earlier onset of colitis, and CD4^+^CD62L^−^ cells from germ free mice were not able to ameliorate colitis compared with mice reconstituted with lymphocytes from conventionally housed animals [22]. The study also assumed a lack of Treg cells within germ free mice by observing the higher percentage of CD4^+^GITR^+^ expressing lymphocytes and the production of IL-10 after priming by dendritic cells, which suggested the presence of Treg cells within the CD4^+^CD62L^+^ lymphocyte subset derived from conventional housed mice. Butyrate, produced by commensal microorganisms during starch fermentation, may facilitate extrathymic generation of Treg cells [23].

### 2.2. Gut Bacteria Benefit the Host

Not only do gut bacteria benefit the host by contributing to the host gut defense system, they also help the gut to maintain normal functions. Gut bacteria benefit the host in a variety of ways, such as regulating gut motility, producing vitamins, transforming bile acid and steroids, metabolizing xenobiotic substances, absorbing minerals, and activating and destroying toxins, genotoxins, and mutagens [24]. The proximal region of colon produces a great quantity of short-chain organic acids, such as acetic, propionic, and butyric acids. Theses organic acids are energy sources for the colonic mucosa and peripheral body tissues, and they are metabolites of undigested complex carbohydrates by colonic bacteria fermentation. In return, theses organic acids affect bacterial growth in the colon by affecting colonic water absorption and decreasing fecal pH. In addition, *Oxalibacterium formigenes*, a betaproteobacterium within the order *Burkholderiales*, which is among the putative core bacteria, is one of the few colonic bacteria with well-defined health benefits. They regulate the homeostasis of oxalic acid and prevent the formation of kidney stones [25].

Gut bacteria are essential for the transformation of natural compounds (e.g., lignans) to perform their bioactivities. Lignans are present in a wide range of foods, such as flaxseed, vegetable, fruit, and beverages. Lignans afford protection against cardiovascular diseases, hyperlipidemia, breast cancer, colon cancer, prostate cancer, osteoporosis and menopausal syndrome, dependent on the bioactivation of these compounds to enterolactone (ENL) and enterodiol [26,27]. Gut bacteria are required for the production and bioavailability of these enterolignans. Secoisolariciresinol is one of the most abundant dietary lignans, and it can be demethylated and dehydroxylated by two gut bacteria isolated from human feces, named *Peptostre ptococcus* SECO-Mt75m3 and *Eggerthella lenta* SECO-Mt75m2 [28]. Gut bacteria also play an essential role in the metabolism of isoflavones, and the metabolites are more biologically active than their precursors. Isoflavones are structurally similar to the mammalian estrogen, and soy foods are the predominant food sources of them. Isoflavones have protective activity in breast cancer, prostate cancer, cardiovascular disease, osteoporosis, and menopausal symptoms [29]. In addition, De Fillippo *et al.* [14] reported that gut bacteria protected African children from the risk of infectious and noninfectious colonic diseases by coevolving with the polysaccharide-rich diet, which also allowed them to maximize energy intake from fibers.

### 2.3. Dietary Influence on Gut Bacteria

**Figure 2:**
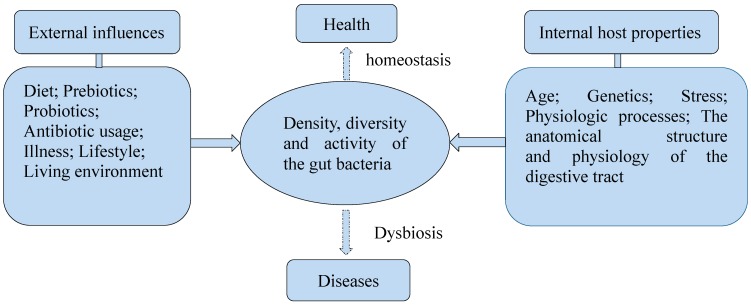
Several factors influence the density, diversity, and activity of the gut bacteria.

The colonization of gut bacteria is influenced by many factors, such as the living environment and diet (Figure 2). The feeding ways of infants was reported to impact the composition of gut bacteria. Infants fed with breast milk had higher levels of *Bifidobacteria* spp*.*, while infants fed with formula had higher levels of *Bacteroides* spp., *Clostridium coccoides* and *Lactobacillus* spp. [30,31,32]. Besides, the host physiologic process, the anatomical structure and physiology of the digestive tract are major factors [24]. They may cause changes to the disease structure in the host. It was proved that diets could impact the composition of gut bacteria. A study showed that mice fed with Western-diet and low-fat-chow-diet displayed different structures of gut bacteria. The relative abundance was increased about 1.2-fold for *Bacteroidetes* and 18-fold for *Proteobacteria*, while was decreased about 1.5-fold for *Firmicutes* in mice fed with Western-diet. Members of the *Desulfovibrionaceae* family were significantly enriched in the cecal contents of healthy mice fed with Western-diet. *Lactobacillus gasseri* species were found representing 4.3% of total bacteria on average, and *Ruminococcus* and other members of *Lachnospiraceae* and *Bacteroidales* were also enriched in mice fed with low-fat-chow-diet. *Lactobacillus gasseri* species were even absent in mice receiving Western-diet [33]. There are also studies reporting that long-term and short-term diets influence the composition and function of the gut microbiota in humans. In a study of diet inventories and 16S rDNA sequencing to characterize fecal samples from 98 individuals, enterotypes were strongly associated with long-term diets, particularly protein and animal fat (*Bacteroides*) versus carbohydrates (*Prevotella*). Microbiome composition changed detectably within 24 h of initiating a high-fat/low-fiber or low-fat/high-fiber diet, but that enterotype identity remained stable during the 10-days in a controlled-feeding study of 10 subjects [34]. Another study showed the short-term consumption of diets composed entirely of animal meat, eggs, and cheeses or plant rich in grains, legumes, fruits, and vegetables, altered microbial community structure and overwhelmed inter-individual differences in microbial gene expression. The animal-based diet increased the abundance of bile-tolerant microorganisms and decreased the levels of *Firmicutes* that metabolize dietary plant polysaccharides. The bile-tolerant microorganisms included *Alistipes*, *Bilophila*, and *Bacteroides*, and *Firmicutes* included *Roseburia*, *Eubacterium rectale*, and *Ruminococcus bromii*. Both diets also altered microbial metabolic activity. The animal-based diet resulted in significantly lower levels of the products of carbohydrate fermentation and a higher concentration of the products of amino acid fermentation compared with the plant-based diet and baseline samples [35].

Further study found that dietary factors such as polyphenols, fibers, and carbohydrates had the ability to modify the balance of gut bacteria. Phenolic acids and flavonoids are the main polyphenols of our dietary intake. It was shown that tea phenolics and their derivatives repressed the growth of certain pathogenic bacteria such as *Clostridium perfringens*, *Clostridium difficile*, and *Bacteroides* spp., while they less severely affected commensal anaerobes, such as *Clostridium* spp., *Bifidobacterium* spp., and *Lactobacillus* sp*.* [36]. Parkar *et al.* [37] found dietary polyphenols may modify the gut bacteria balance indirectly by their biotransformation products rather than the original plant compounds. In that study, fermentation of polyphenols increased proliferation of *Bifidobacteria* and decreased the ratio of *Firmicutes* to *Bacteroidetes*, compared to controls. Furthermore, among the polyphenols studied, caffeic acid at 100 mg/mL stimulated the greatest absolute increases of gut bacteria. Polyphenols also stimulated the production of short chain organic acids by the gut bacteria. Fiber was another dietary factor that impacted the composition of gut bacteria. A study showed that subjects on a fiber-blend fortified enteral formula had less negative symptoms related to bowel urgency, and decreases in total bacteria and *Bifidobacteria* were less severe compared with the fiber-free formula [38]. Polyphenols and fibers were thought to be beneficial dietary factors. Functional foods based on these beneficial dietary factors may provide opportunities to modulate the bacteria balance in the gut. In addition, a recent study showed the pH of drinking water could also affect the composition and diversity of commensal bacteria in the gut [39].

However, some dietary factors may be harmful, such as dietary iron. Dietary iron mostly from red meat and fortified cereals can also change the gut bacteria composition. Other luminal iron is from cigarette smoking. Increased iron availability may increase the proliferation and virulence of gut bacteria and increase the permeability of the gut barrier. A study showed that increased iron exposure contributed to the colonization of certain bacterial pathogens including *Salmonella* [40]. It may be a risk factor for colorectal cancer. The composition of intestinal bacteria could also be regulated by traditional Chinese herbs. The five hydroxyanthraquinone derivatives from *Rheum palmatum* had inhibitive effects on *Bifidobacterium adolescentis* growth [41]. The most effective component in *R. palmatum* to restrain the growth of *B. adolescentis* was rhein.

It was proved that prebiotics could influence the composition of gut bacteria to benefit the host. Prebiotics are defined as a non-digestible food ingredient that beneficially affects the host by selectively stimulating the growth and/or activity of one or a limited number of bacteria in the colon, and thus improving the hosts’ health [42]. Prebiotics are carbohydrate-like compounds, such as lactulose and resistant starch, and have been used in the food industry to modify the composition of the microbiota species to benefit human health in recent years [43]. Inulin is one type of prebiotics. These prebiotics mostly target bifidobacteria and lactobacilli, which are two kinds of probiotics [44]. Probiotics are living non-pathogenic organisms used as food ingredients to benefit the hosts’ health. They may be lactic acid bacteria, *Bifidobacteria*, or yeasts, such as *Saccharomyces cerevisiae* [45,46]. Furthermore, probiotics can be used in the treatment of hepatic encephalopathy, inflammatory bowel diseases, infections, hypertension, cancer, and atopic dermatitis in children [46,47,48,49,50].

## 3. Gut Bacteria and Diseases

Usually, gut bacteria and the host live in a commensal manner. However, gut bacteria can be potentially harmful when the ecosystem undergoes abnormal changes. Dysbiosis of the gut bacteria communities in patients or animal models may cause many diseases. For example, antibiotic treatment and surgery cause pseudomembranous colitis due to toxin production by *Clostridium difficile* and sepsis of *Escherichia coli*, *Enterococcus faecalis* and *Enterococcus faecium*, and intra-abdominal abscesses due to *Bacteroides fragilis* [51]. Imbalance in gut bacteria composition was associated with intestinal symptoms, such as bloating, abdominal pain, and diarrhoea. Uncultured phylotypes from *Clostridium* clusters IV and XIVa had statistically significant positive correlation with bloating. *Anaerotruncus colihominis*, *Ruminococcus callidus* and *Lachnospira pectinoschiza* were higher when bloating was recorded. The abundance of bifidobacteria may have a negative correlation with abdominal pain. In addition to bifidobacteria, one phylotype within *Ruminococcus lactaris*, *Clostridium* cluster IV, was significantly decreased in pain associated samples. Among the positive correlations, uncultured, potentially pathogenic phylotypes within uncultured *Clostridiales* II, *Anaerotruncus colihominis* and *Ruminococcus callidus*, were increased over ten-fold when pain was recorded [25]. Diarrhea was associated with a reduced number of members from the genus *Streptococcus*, particularly *S. alactolyticus* [15]. Besides, many other diseases are related to gut bacteria, such as inflammatory bowel diseases, obesity, diabetes, liver diseases, chronic heart diseases, cancers, HIV, and autism.

### 3.1. Gut Bacteria and Inflammatory Bowel Diseases

Inflammatory bowel disease (IBD) is most common in the developed countries of Europe, the U.S., and Scandinavia [24]. Reciprocal interaction between commensal gut bacteria and the host may induce allergies and IBD. Overly aggressive Th-1 mediated cytokine response to commensal bacteria may be the pathogen of chronic intestinal inflammation [52]. In addition, disorders in bacterial recognition by macrophages are strongly related to pathogenesis of IBD. Furthermore, IBD could results from an abnormal immune response against the commensal microbiota in a genetically susceptible host. Jostins *et al.* [53] have identified 163 risk loci associated with IBD, and found that many loci were involved in the sensing and elimination of bacteria. A hypothesis is that the innate immune system in IBD patients could be deficient, which in turn leads to an uncontrolled adaptive response.

Ulcerative colitis (UC) is one of the two major idiopathic IBDs [54]. In UC patients, the disease is limited to the colon. Numbers of lactobacilli were significantly lower during the active phase of the disease, and denaturing gradient gel electrophoresis analysis suggested that *Lactobacillus salivarus*, *Lactobacillus manihotivorans* and *Pediococcus acidilactici* were present in remission, but not during active inflammation [55]. Besides, the colonic bacterial communities from diseased mice were less complex, indicating less diversity of bacterial composition during acute inflammation. Bacteria of the *Clostridiales* group were more prominent in samples from the inflamed colon, indicating these bacteria might accumulate during colitis [56]. In a study of an animal colitis model, *E. coli* may have served as a biomarker for colitis severity. The development of colitis is associated with higher *E. coli* loads, and bacterial TLR2 ligands may contribute to colitis pathology. Bacterial products exacerbate acute inflammation via TLR2- and TLR4-signaling and potentially trigger TLR-dependent accumulation of neutrophiles and T-cells. IL-10^−/−^ mice developed Th1-dominant colitis via IL-12 and IL-23 hyperproduction through bacteria recognition by abnormally differentiated subsets of intestinal macrophages [57]. A recent study observed a reduction of *Roseburia hominis* and *Faecalibacterium prausnitzii* in fecal samples of UC patients, and both species showed an inverse correlation with disease activity [58]. UC patients had different gene expression profiles and lower levels of biodiversity than their healthy twins, as well as unusual aerobic bacteria. They also had lower percentages of potentially protective bacterial species (e.g., *Lachnospiraceae* and *Ruminococcaceae* families) than their healthy twins. The colonic microbiota appeared to interact with the transcriptional profile of the mucosa. This interaction appeared to be lost in the colon of UC patients. Bacterial functions, such as butyrate production, might affect mucosal gene expression [59]. It has been suggested that prebiotic combination (a combination of chicory-derived long-chain inulin and oligofructose) reduced colitis in HLA-B27 transgenic rats, associating with alterations to the gut bacteria, decreased proinflammatory cytokines, and increased immunomodulatory molecules [52].

Crohn’s disease (CD) is another type of IBDs. It has been thought to be an autoimmune disease, in which the body’s immune system attacks the gastrointestinal tract and causes inflammation [60]. Seksik *et al.* [61] found that the fecal microflora in patients with both inactive and active colonic CD contained significantly more enterobacteria than in healthy subjects. In addition, about 30% of the dominant bacteria did not belong to the usual dominant phylogenetic groups. Another study found that five bacterial species characterised dysbiosis in CD patients, which were a decrease in *Dialister invisus*, an uncharacterised species of *Clostridium* cluster XIVa, *Faecalibacterium prausnitzii* and *Bifidobacterium adolescentis*, and an increase in *Ruminococcus gnavus*. There was a different composition of gut microbiota in unaffected relatives of patients with CD compared with healthy controls. This dysbiosis was not characterized by lack of butyrate producing-bacteria as observed in CD but suggested mucin-degradation capacity of microorganisms [62]. In addition, an increased abundance in bacteria including *Enterobacteriaceae*, *Pasteurellacaea*, *Veillonellaceae*, and *Fusobacteriaceae* and decreased abundance in *Erysipelotrichales*, *Bacteroidales*, and *Clostridiales*, correlated strongly with disease status in a large pediatric CD cohort study. The study also indicated that antibiotic use amplified the microbial dysbiosis associated with CD. It suggested as well that assessing the rectal mucosal-associated microbiome offered unique potential for convenient and early diagnosis of CD at this early stage of disease [63]. Exclusive enteral nutrition treatment significantly changed gut bacterial composition, and the changes of gut *Bacteroides* species were associated with reduced inflammation in CD patients [64]. However, resident *Bifidobacteria* and *Lactobacilli* did not suppress the growth of disease-inducing *Bacteroides* species or clostridia to mediate protective action. Furthermore, several studies showed that prebiotic and probiotic use has an effect on the induction of remission in IBD patients [65]. In general, gut bacteria take a critical role in the development of IBD through the regulation of inflammation in the gut.

### 3.2. Gut Bacteria and Obesity

Normal gut bacteria play an important role in diet-induced obesity, because germ-free mice have been reported to be thinner and did not become adipose when subjected to high-fat diet [66]. The high-fat diet altered the composition of bacteria to display higher levels of luminal *Firmicutes* and *Proteobacteria* and lower levels of *Bacteroidetes* [67], indicating that obesity may be associated with decreased diversity and changes in composition of the gut bacteria. Gut bacteria is an important determinant of susceptibility to obesity and related metabolic diseases. The ratio of *Firmicutes* to *Bacteroides* has been found to be correlated to body weight, with the ratio being higher in obese people [68]. Gut bacteria could also affect obesity by promoting chronic inflammatory status [69]. In addition, *Clostridium difficile* infections may be another possibility of causing obesity [70]. Gut bacteria may affect obesity through regulation of the microbiota-brain-gut axis by its metabolites. Overweight individuals have more faecal SCFAs than lean individuals, with significantly increased levels of propionate. Butyrate and propionate were reported to protect against diet-induced obesity and regulate gut hormones via free fatty acid receptor 3(FFAR3)-independent mechanisms in mice [71]. SCFAs (propionate and butyrate) activated intestinal gluconeogenesis (IGN) by complementary mechanisms. Butyrate directly activated IGN gene expression via an increase in cAMP in enterocytes. Propionate, acting as a FFAR3 agonist in the periportal afferent neural system, induced IGN via the microbiota-gut-brain axis. A portal vein glucose sensor detected glucose produced by IGN and communicated to the brain by the peripheral nervous system to promote beneficial effects on food intake and glucose metabolism [72].

Conjugated linoleic acid (CLA) has anti-obesity effect. Some rumen bacteria have the ability to form CLA from diets, such as food products from beef, milk fat, natural, and processed cheeses, yogurt, and plant oil. The amount of CLA in human adipose tissue is thought to be directly related to dietary intake. It was found that six strains (four *Bifidobacterium breve* strains, a *Bifidobacterium bifidum* strain and a *Bifidobacterium pseudolongum* strain) were able to produce different CLA and conjugated α-linolenic acid isomers from free linoleic acid and α-linolenic acid [73]. *Lactobacillus rhamnosus* PL60 is a human originated bacterium that produces t10, c12-CLA. A study showed that after eight weeks of feeding, *L. rhamnosus* PL60 reduced the body weight of diet-induced obese mice without reducing energy intake, and caused a significant, specific reduction of white adipose tissue, including epididymal and perirenal [74]. There was no observation of liver steatosis, a well known side effect of CLA by *L. rhamnosus* PL60 treatment. Furthermore, oral *Lactobacillus reuteri* therapy alone prevented the pathology of abdominal obesity and age-associated weight gain in mice regardless of their baseline diet, and changed the pro-inflammatory immune cell profile, which was particularly dependent on CD4^+^ T cells and the presence of anti-inflammatory IL-10 [75]. In addition, an epidemiological study showed that eating yogurt surprisingly prevented age-associated weight gain, which may be due to a probiotic bacteria-mediated mechanism [76]. Dietary probiotic consumption alters gut bacteria, which may affect not only intestinal health but also distant tissues and overall health and longevity by immune-mediated mechanisms. Prebiotics and probiotics can also be used as a combination, namely synbiotics, to fight obesity [77,78]. Furthermore, the microbiota can also be manipulated through fecal microbiota transplantation (FMT). Animals who received a transplant from the obese (Ob) twin donors developed increased body mass and adiposity compared to those receiving transplants from lean (Ln) twin donors. Cohousing Ln and Ob mice prevented the development of increased adiposity in Ob cage mates and transformed their microbiota’s metabolic profile to a lean-like state. However, the microbial protection from increased adiposity was only possible against the backdrop of a suitable host diet [79].

Besides probiotics, other gut bacteria could also protect humans from obesity. *Bacteroidetes phylum*, particularly *Bacteroides* spp., was suggested to be mainly responsible for protection against increased adiposity [80]. More complex bacterial interactions and associated metabolic disturbance were involved in protection against increased adiposity. The study also showed that microbial protection from increased body mass was only possible when the hosts have a suitable diet. In addition, a study showed that mice were protected against high-fat diet induced obesity by modified *E. coli* Nissle 1917 expressing either *N*-acylethanolamines or their precursors [81]. Therefore, targeting gut bacteria may provide a novel way for the treatment of obesity.

### 3.3. Gut Bacteria and Diabetes

High-fat diet induced obesity is associated with inflammation that contributes to the development of insulin resistance, which may cause type-2 diabetes (T2D). Diabetes (a metabolic disease) has been a big problem all over the world, and it has been shown to be strongly associated with gut bacteria. There are two types of diabetes, type-1 diabetes (T1D) and T2D. Changes in the gut bacteria contribute to diabetes. The occurrence of diabetes is impacted by early intestinal microbial colonization at birth, which is affected by the feeding ways, birth weight, and the delivery method at birth [82]. In addition, LPS was thought to be a novel factor triggering the onset of high-fat diet-induced T2D [83].

T1D is a destructive islet β-cell specific autoimmune disease with loss of ability of insulin production, which results from interaction between genetic and environmental factors. Gut bacteria manipulation modulated mucosal oxidative stress and pro/anti-inflammatory balance to protect against T1D, and eventually restored the intestinal mucosal barrier function [84]. Impaired intestinal mucosal barrier and altered mucosal immunity were involved in the pathogenesis T1D. Aberrant gut bacteria may contribute to the pathogenesis of T1D, since cross-talk between gut bacteria and the innate immune system may be involved in islet destruction [85].

Mining 16S rRNA data showed a lower proportion of butyrate-producing and mucin-degrading bacteria, while those bacteria that produce short chain organic acids other than butyrate were higher in T1D cases compared to controls [86]. Butyrate and mucin are essential to maintain gut integrity. Murri *et al.* [87] found the number of *Clostridium*, *Bacteroides* and *Veillonella* significantly increased, and the number of *Lactobacillus*, *Bifidobacterium*, *Blautia coccoides*/*Eubacterium rectale* group and *Prevotella* significantly decreased in children with T1D. They also found that the number of *Bifidobacterium*, *Lactobacillus*, and the *Firmicutes* to *Bacteroidetes* ratio had a significantly negative correlation with the plasma glucose level while the quantity of *Clostridium* had a significantly positive correlation with the plasma glucose level in the diabetes group. However, another study found class *Clostridia* were significantly reduced in the T2D compared to the control. The phylum *Firmicutes* was also reduced. In addition, the ratios of *Bacteroidetes* to *Firmicutes* had a significantly positive correlation with plasma glucose concentration, which was in agreement with Murri *et al.* [87]. The ratios of *Bacteroides*-*Prevotella* group to *C. coccoide*-*E. rectale* group also had a positive correlation with plasma glucose concentration. Class *Betaproteobacteria* was highly increased in diabetics compared to the non-diabetic and positively correlated with plasma glucose [88].

*Bifidobacterium* spp*.* had a significantly negative correlation with endotoxaemia, which was associated with the mechanism of diabetes and/or obesity. Meanwhile, *Bifidobacterium* spp*.* had a significantly positive correlation with improved glucose tolerance, glucose-induced insulin secretion and normalized inflammatory tone with high-fat prebiotic treated mice [83]. Another study showed that *Bifidobacterium animalis* reversed metabolic bacteremia which was caused by translocation of bacteria from the intestine to the mesenteric adipose tissue and the blood during the early onset of a high-fat diet-induced T2D. The treatment improved the animals’ overall inflammatory and metabolic status, since glucose intolerance was moderately blunted, and insulin sensitivity and fasting hyperinsulinemia were completely normalized [89]. In addition, antibiotic therapy had a positive result in animal diabetic models. A study showed that the frequency of diabetes in female non-obese diabetic mice provided with chow containing doxycycline was significantly lower than in animals provided with standard chow [90].

### 3.4. Gut Bacteria and Liver Diseases

The gut and liver have a close interplay based on the evidence that the gut absorbs beneficial substances produced by the liver. The liver receives approximately 70% of its blood supply from the intestinal venous outflow, which represents the first line of defense against gut-derived antigens and is equipped with a broad array of immune cells, including macrophages, lymphocytes, natural killer cells, and dendritic cells, to accomplish this function [91]. Gut bacteria play a key role in the maintenance of gut-liver axis health. Ethanol, ammonia, and acetaldehyde produced by the intestinal microflora are generally metabolized by the liver and control Kupffer cell activity and cytokine production.

Small intestinal bacteria overgrowth (SIBO) may be an important pathogenesis of nonalcoholic steatohepatitis (NASH). Small intestinal movement was decreased by small intestinal bacteria overgrowth in NASH rats. Antibacterial treatment can alleviate the severity of NASH [92]. In the study, *E. coli* was excessively increased in NASH rats, as was the serum level of aminopherase. TNF-α may be an important mediator in the promotion of NASH by SIBO, which was supported by the fact that the level of aminopherase went up and down with the serum level of TNF-α. Nevertheless, another study suggested the severity of Concanavalin-A (ConA) induced hepatitis was increased when intestinal bacterial flora were suppressed by antibiotics. Reconstitution of intestinal flora with H_2_-producing *E. coli* alleviated the ConA-induced liver inflammation. But H_2_-deficientmutant *E. coli* did not have this effect. These results suggested that H_2_ released from intestinal bacteria can suppress ConA induced inflammation in liver [93]. Recent evidence [94] also indicated that the gut microbiota is associated with alcoholic associated liver damage. Gut-derived endotoxin and other luminal bacterial products may be cofactors for the development of alcoholic liver disease. Daily alcohol consumption could affect the composition of colonic microbiome, which suggests that dysbiosis in the gut bacteria communities may be an important mechanism of alcohol-induced endotoxemia [94].

Many hepatitis patients could develop liver cirrhosis, which is an asymptomatic process. The variations in quantitative and qualitative intestinal bacteria lead to an increase in intestinal permeability and endotoxin translocation. Consequently, the action induces the transcriptional activation of quite a lot of pro-inflammatory genes and cytokines in the liver. Translocated intestinal bacteria could cause spontaneous bacterial peritonitis in cirrhotic rats, which aggravated cirrhosis [95]. Hepatic encephalopathy (HE) is a common and dreaded complication of liver disease. There are urease-positive bacteria that can produce ammonia from aminoacids through de-amination, which is an important critical factor in pathogenesis of HE. Treating HE with probiotics showed to be more effective than prebiotics and antibiotics [46].

### 3.5. Gut Bacteria and Chronic Heart Diseases

Gut bacteria have a direct link with the risk of cardiovascular diseases. They form trimethylamine (TMA) from dietary choline after its conversion into TMA N-oxide in the liver [96]. TMA could act as a pro-atherogenic compound. Intestinal concentration of mostly adherent bacteria in chronic heart failure (CHF) patients increased, and adherent invasive *Escherichia coli* were identified [97]. Disturbed intestinal microcirculation and barrier function in chronic heart disease likely trigger cytokine generation, contributing to further impairment in cardiac function. Myocardial dysfunction can lead to microcirculatory injuries reducing a disruption in the intestinal barrier which amplifies the inflammatory response [98]. Colonization with *Lactobacillus brevis* decreased bowel permeability, whereas colonization with *Escherichia coli*, *Klebsiella pneumonia*, and *Streptococcus viridians* showed the opposite effect. It was suggested that a bowel wall with high permeability may lead to bacteria and/or endotoxin translocation, which may be an important stimulus for inflammatory cytokine to be activated in chronic heart failure [99].

Hypercholesterolaemia plays a key role in the development and progression of coronary artery disease. Three *Lactobacillus plantarum* strains (CECT 7527, 7528, and 7529) showed a high ability to survive under gastrointestinal tract conditions and to adhere to intestinal cells with a great production of bile salt hydrolase, especially when combined [100]. Furthermore, the strains assimilated cholesterol directly from the medium. The three strains, especially CECT 7529, produced large quantities of propionic and butyric acids. Dahl S rats fed the commercially available probiotic product Goodbelly, which contains the leptin-suppressing bacteria *Lactobacillus plantarum* 299v, resulted in decreased circulating leptin levels, smaller myocardial infarcts, and greater recovery of postischemic mechanical function [101]. The effects were similar to the effects in rats treated with minimally absorbed antibiotic vancomycin. These results show that *Lactobacillus plantarum* strains may be beneficial for cardiovascular diseases. In addition, gut bacteria has been suggested to play a key role in the oral pharmacokinetics of baicalin, which is a flavonoid purified from *Scutellaria baicalensis* Georgi that has been used for treatment of hypertension, cardiovascular diseases, and viral hepatitis [102].

### 3.6. Gut Bacteria and Cancers

The presence of microbial pathogens or a disorder in the native intestinal bacterial community contributes to the development of cancers, such as gastrointestinal cancer and prostate cancer. Commensal bacteria were recognized as important cofactors in the carcinogenesis of colon. It was reported that gut bacteria can trigger macrophages to produce diffusible clastogens, or chromosome-breaking factors. This action was through a bystander effect which mediated DNA damage and induced chromosomal instability in neighboring cells. In addition, peroxidative stress may play an important role in ileal and colonic pathology and inflammation in bacteria-associated intestinal cancers [103].

The composition of the gut bacteria community is different between healthy individuals and colon cancer patients. Several butyrate-producing bacterial genera were under-represented in the stool of colorectal cancer patients compared to healthy individuals. Two of the *Prevotella* species identified were completely absent from the colon cancer samples analyzed. *Prevotella* was hypothesized to help maximize energy harvest from a plant-based diet. The higher levels of *Prevotella* in the healthy cohort may reflect differences in the intake of fiber and other plant compounds compared to the individuals with colon cancer. On the other hand, *Acidaminobacter*, *Phascolarctobacterium*, *Citrobacter farmer*, and *Akkermansia muciniphila* significantly over-represented in colorectal cancer (CRC) stool samples [104]. *Akkermansia muciniphila* are mucin-degrading species. These may influence the quantities of metabolites in the intestinal tract. Butyric acid was significantly lower in the faeces of colon cancer patients, since species of butyrate producing bacteria (such as *Ruminococcus* spp*.* and *Pseudobutyrivibrio ruminis*) were lower in stool samples from CRC patients compared to healthy controls. Butyrate is quite an important nutrient for normal colon cells, which was shown to reduce proliferation and induce apoptosis of human colon carcinomas cells alone or in combination with propionate. In another study, over 26 novel species assigned to the *Helicobacter genus* (more than 90% similarity) have been identified, only some of which have been directly associated with gastrointestinal cancers [105].

Dietary administration of *Bifidobacterium longum* had significant suppression of colon tumor incidence, tumor multiplicity and tumor volume. It was suggested that oral supplement of *B. longum* exerted strong antitumor activity, as modulation of the intermediate biomarkers of colon cancer indicated antimutagenic effects [106]. In addition, ingestion of *B. longum* has been showed to inhibit azoxymethane-induced cell proliferation, ornithine decarboxylase activity and expression of ras-p21 oncoprotein activity significantly.

Gut bacteria also play an important role in the anticancer immune response. It was demonstrated that cyclophosphamide altered the composition of gut bacteria in the small intestine of mouse models and induced the translocation of selected species of Gram positive bacteria into secondary lymphoid organs, where these bacteria stimulate the generation of a specific subset of “pathogenic” T helper 17 (pTh17) cells and memory Th1 immune responses. Tumor-bearing mice which were germ-free or that had been treated with antibiotics to kill Gram positive bacteria showed a reduction in pTh17 responses and their tumors were resistant to cyclophosphamide. The results suggested that the gut bacteria may help shape the anticancer immune response [107].

Gut bacteria are not only strongly related to gastrointestinal cancer but also prostate cancer. Systemic inflammation triggered by pathogenic gut bacteria played a pivotal role in prostate cancer development. Poutahidis *et al.* [108] found that *Helicobacter hepaticus* infected Apc^Min/+^ mice had significantly increased incidence of prostate intraepithelial neoplasia and microinvasive adenocarcinoma lesions in the prostate. However, the mice did not have overt IBD or large adenomatous polyps in their bowl. The study also assumed that TNF-α and proteases from mast cells residing in the prostate stroma enhanced the carcinogenic process. In addition, neoplasia was transmissible to uninfected mice by receiving only lymph node cells from *H. hepaticus*-infected mutant mice. Commensal intestinal bacteria can be beneficial for prostate cancer with ingestion of a healthy diet. The significant difference in the incidence rate of prostate cancer among Asian and European/North American populations may due to the intake of soy-derived food products and the metabolites of the isoflavones they contain by gut bacteria fermentation [14,109].

### 3.7. Gut Bacteria and HIV

The recent hypothesis is that microbial alterations at gastrointestinal tract level play a key role in the pathogenesis of chronic HIV infection [110]. It was found that gut bacteria of HIV/AIDS patients shared more than 90% sequences of HIV-1. These bacteria were mostly specified as *E. coli* (negative in serotypization), *Proteus mirabilis*, *Citrobacter freundii*, *Staphylococcus* sp*.* and *Enterobacter aerogenes* [111]. These results provided hypothesis that gut bacteria are involved in the pathogenesis of HIV. HIV infection results in deterioration of gut homeostasis, which leads to increased bacterial compounds in the circulation. These bacterial components including LPS, peptidoglycan, and bacterial DNA may further stimulate the vicious circle of immune activation, which in turn contributes to viral replication and the progression of disease [110]. Imbalance of the intestinal immune barrier, translocation of immunostimulatory microbial products, and chronic systemic inflammation was thought to drive HIV infection to AIDS.

Vujkovic-Cvijin *et al.* [112] found that 579 taxa were enriched and 45 taxa were depleted in viremic untreated (VU) HIV-infected subjects compared to HIV^−^ subject samples. The most enriched taxon was *Erysipelotrichaceae* in the class *Mollicutes*, which has been associated with obesity and heightened cardiovascular morbidity. Members of the phylum *Proteobacteria* were included in the most enriched taxa in VU subjects. Enriched genera from the *Enterobacteriaceae* family included *Salmonella*, *Escherichia*, *Serratia*, *Shigella*, and *Klebsiella* species, which were known as pro-inflammatory pathobionts. Additionally, *Staphylococcus*, *Pseudomonas*, and *Campylobacter* spp*.* were highly enriched in the mucosae of VU subjects, and they are known as opportunistic pathogens and sources of bacteremia in HIV-infected subjects. The relative abundance of specific members of *Clostridia* and *Bacteroidia* were significantly reduced in VU subjects, with the greatest degree of depletion among members of the *Bacteroides* and *Alistipes genera*. More importantly, the study also found that HIV-infected subjects seemed to harbor the enteropathogenic bacteria community that can catabolize tryptophan into immunomodulatory kynurenine derivatives, which is known to correlate with the progression of disease and contribute to mucosal immune disruption.

Patients living with HIV showed a dramatic decline of lactobacilli and bifidobacteria and a higher concentration of pathogenic species including *Candida albicans* and *Pseudomonas aeruginosa* [113,114]. Probiotics such as *Lactobacillus rhamnosus* GR-1 have significant promise in supporting the immune function of people living with HIV [115]. Gori *et al.* [110] found that *bifidobacteria* increased significantly, with decreasing *Clostridium coccoides*, *Eubacterium rectale*, *Clostridium lituseburense*, and *Clostridium histolyticum* when prebiotics were supplied. The study also found dietary supplementation with a prebiotic oligosaccharide mixture reduced sCD14, CD4^+^ T-cell activation (CD25), and improved NK cell activity in highly active antiretroviral therapy-naive HIV-1 infected adults.

### 3.8. Gut Bacteria and Autism

Autism spectrum disorders (ASDs) are neurodevelopmental disorders, which are characterized as cognitive impairments, stereotyped behaviors, and impairments in social skills. ASDs include autism, Asperger’s syndrome, and pervasive developmental disorder not otherwise specified (commonly abbreviated as PDD-NOS). The number of people diagnosed with autism has been increasing dramatically since the 1980s. Autism has been thought to be linked to gastrointestinal symptoms, such as diarrhea, combined with genetic predisposition and environmental factors [116]. There might be a gut-brain axis in the pathogenesis of autism. Gut bacteria possibly communicate with the central nervous system through neural, endocrine and immune pathways to influence brain function and behavior [117]. Several intestinal bacteria are involved in the pathogenesis of autism. Individuals with ASDs responded well to the antibiotics vancomycin and metronidazole, although vancomycin is not absorbed from the gastrointestinal tract. Overgrowth of *Clostridia* and a decrease in *Bifidobacteria* could be involved in ASD pathogenesis [118], as clostridium produces exotoxins and propionate, the latter worsened ASD-like behavior.

Autistic (AD) children have a distinct and less diverse gut microbial community structure, and showed significantly lower levels of genera *Prevotella*, *Coprococcus*, and unclassified *Veillonellaceae* [119]. Two organisms (*Bacteroides vulgatus* and *Desulfovibrio* species, including *D. desulfuricans*, *D. fairfieldensis*, and *D. piger*) were more commonly found in stools of AD children than in the control children’s stools. *Firmicutes* and *Actinobacteria* accounted for less of the total flora of AD children’s stools than the control children’s stools [120]. The most striking finding was that significant numbers of non-spore-forming anaerobes and microaerophilic bacteria were found in gastric and duodenal specimens from children with autism, while such bacteria are totally absent in the gut and duodenum from control children [121]. Another study also suggested that *Desulfovibrio* was more common in AD children than in controls. In addition, siblings of AD children had intermediate counts of *Desulfovibrio*, suggesting possible spread of the organism in the family environment [122].

Based on 16S-rRNA and culture-dependent data, De Angelis *et al.* [123] found *Caloramator*, *Sarcina* and *Clostridium genera* were higher in AD children compared with healthy children. The composition of *Lachnospiraceae* family also differed in AD children. The level of *Eubacteriaceae* in fecal samples of AD children was lower, except for *Eubacterium siraeum*. The levels of some *Alistipes* and *Akkermansia* species as well as almost all the identified *Sutterellaceae* and *Enterobacteriaceae* were also higher in AD children. If the differences of gut bacteria between AD and healthy children are one of the causes or the consequence of autism, they may be clues for a specific diagnostic test, and/or for prevention and treatment.

### 3.9. Gut Bacteria and Other Diseases

Gut bacteria are also related with several other diseases and malaise, such as bad sleep, rheumatic diseases, and kidney diseases. Sleep in functional constipation subjects may be worse than that in control subjects, that is to say wake after sleep onset (WASO) and WASO (%) (WASO/total sleep time multiplied by 100) in functional constipation patients were longer and greater. The study also found *Bifidobacterium* counts per gram of wet stools and proportion in total bacterial cell counts were significantly lower in functional constipation patients [124]. Bad sleep, functional constipation, and low *Bifidobacterium* proportion may have some connection.

The relationship between rheumatic diseases (RA) and microbial components has been elucidated. It was found that joint inflammation did not develop in germ-free conditions in animal models of human spondylarthropathy in HLA-B27 transgenic rats or in B10.BR mice [125,126]. Rheumatic arthritis patients had significantly reduced fecal carriage of *Bifidobacteria* and *Bacteroides fragilis*. Obesity may be a factor in the aetiology of RA. Increased LPS uptake through the gut lumen to other tissues occurs in obese murine models, and enhanced systemic exposure to LPS could increase the risk of RA [127].

In addition, intestinal bacteria may be involved in chronic kidney disease. It was assumed that intestinal bacteria promote the uraemic syndrome by the production of uraemic toxins [128]. Tyrosine or phenylalanine fermentation by intestinal bacteria generates p-cresol, which is circulated mainly as *p*-cresyl sulfate (*p-*CS). The *p-*CS, known as uremic retention solute, accumulates in the blood of chronic kidney disease patients. Composition of microbiota was suggested to influence the production of p-cresol. *Bacteroides fragilis* and *Clostridium difficile* were reported to produce p-cresol *in vitro* [129]. Th1-type cellular immune response, which plays an important role in protection against infectious diseases, was suppressed by intestinal bacteria-derived *p-*CS [130]. Some examples of dysbiosis found in human diseases are shown in Table 1.

**Table 1:** Some examples of dysbiosis found in human diseases.

Disease	Model	Dysbiosis	Sample	References
Ulcerative colitis	Mice	↓*Lactobacilli* ↑*Clostridiales*	Colonic	[56]
	Mice	*↑E. coli*	Colonic	[57]
	Humans	↓*R.* hominis ↓*F. prausnitzii*	Fecal	[58]
Crohn’s disease	Humans	↓*Bacteroides* ↓*Bifidobacteria*	Fecal	[61]
Obesity	Mice	↓*Bacteroides* ↑*Firmicutes* ↑*Proteobacteria*	Fecal	[67]
Type-1diabetes	Humans (children)	↓*Lactobacillus* ↓*Bifidobacterium* ↓*Blautia coccoides* ↓*Eubacterium rectal* ↓*Prevotella* ↑*Clostridium* ↑*Bacteroides* ↑*Veillonella*	Fecal	[87]
Type-2 diabetes	Humans	↓*Clostridia* ↓*Firmicutes* ↑*Betaproteobacteria*	Fecal	[88]
Nonalcoholic steatohepatitis	Rats	↑*E. coli*	Proximal small intestine	[92]
Colorectal cancer	Humans	↓*Prevotella* ↓*Ruminococcus* spp. ↓*Pseudobutyrivibrio ruminis* ↑*Acidaminobacter*, ↑*Phascolarctobacterium*, ↑*Citrobacter farmer* ↑*Akkermansia muciniphila*	Fecal	[104]
HIV	Humans	↑*Erysipelotrichaceae* ↑*Proteobacteria* ↑*Enterobacteriaceae* ↓*Clostridia* ↓*Bacteroidia*	Proctosigmoid	[112]
HIV	Humans	↓*Lactobacilli* ↓*Bifidobacteria* ↑*Candida albicans* ↑*Pseudomonas aeruginosa*	Fecal	[113,114]
Autistic	Humans (children)	↑*Bacteroides vulgates* ↑*Desulfovibrio* ↓*Firmicutes* ↓*Actinobacteria*	Fecal	[122]
Rheumatic arthritis	Humans	↓*Bifidobacteria* ↓*Bacteroides fragilis*	Fecal	[127]

## 4. Conclusions and Prospects

This review provides current understanding of the role of gut bacteria in human health and diseases. Gut bacteria have been found to be involved in many diseases, such as IBD, obesity, diabetes, carcinoma, HIV, and autism. When the gut bacteria undergo some imbalance, several diseases may occur. Immunoregulatory activity is the main function of gut bacteria in the pathogenesis of these diseases. Diet-induced dysbiosis affects disease susceptibility, including IBD, diabetes, and obesity. In recent years, prebiotics and probiotics have been widely used in the treatment of some diseases, and have shown great effects. Fecal microbiota transplant is also a way to modulate gut bacteria. However, there are many questions open, for example, if the changes of gut microbiota are the causes or the consequences of the diseases? Furthermore, some studies have even obtained different results. In order to explore the exact pathogenesis of the gut bacteria related diseases and the role of gut bacteria in these diseases, further studies should be carried out. Butyrate has been shown to be quite an important nutrient for normal colon cells, and could reduce proliferation and induce apoptosis of human colon carcinomas. More studies, therefore, should be carried out to identify butyrate-producing bacteria. In addition, mixed use of prebiotics and probiotics should be further investigated, considering their benefits on human health. Since gut bacteria have important impacts on human health and diseases, they can be used as a novel target to prevent and treat many chronic diseases, and further studies are guaranteed to target them in different ways to fight against gut bacteria-related diseases. Furthermore, special attention should be paid to gut microbiomics to better understand the relationship between gut microbiota and human health, which will provide perspectives for personalized gut microbiota management and bacteriotherapy.
